# Supplementary material for: Ketoprofen Lysine Salt Versus Corticosteroids in Early Outpatient Management of Mild and Moderate COVID-19: A Retrospective Study
Source: Pharmacy (Basel). 2025 May 1;13(3):65. doi: 10.3390/pharmacy13030065 (PMC12101418; doi:10.3390/pharmacy13030065)
Supplement: Supplementary file 1 [file pharmacy-13-00065-s001.zip › Table S1.pdf]

Hospitalization Collinearity

	VIF	Tollerance
Smoking Status	1.07	0.938
Ischemich Heart Disease/CHF	1.00	1.000
Type II Diabetes	1.07	0.934
Haematological Disorders	1.00	1.000
Dyspnea/Chest tightness	1.02	0.981
Therapy Group	1.03	0.973

VIF: Variance inflation factor (VIF)

Requiring Oxygen Sypport Collinearity

	VIF	Tollerance
Smoking Status	1.11	0.899
Ischemich Heart Disease/CHF	1.00	1.000
Type II Diabetes	1.10	0.906
Haematological Disorders	1.00	1.000
Dyspnea/Chest tightness	1.02	0.977
Therapy Group	1.01	0.994

VIF: Variance inflation factor (VIF)

Persistent long COVID symptoms Collinearity

	VIF	Tollerance
Smoking Status	1.04	0.958
Ischemich Heart Disease/CHF	1.11	0.897
Type II Diabetes	1.16	0.862
Haematological Disorders	1.11	0.902
Dyspnea/Chest tightness	1.04	0.959
Therapy Group	1.04	0.957

VIF: Variance inflation factor (VIF)
